# Supplementary material for: Link between bilaterality and type 2 inflammation in respiratory epithelial adenomatoid hamartomas: A two-center retrospective cohort study
Source: PLoS One. 2025 Feb 3;20(2):e0318452. doi: 10.1371/journal.pone.0318452 (PMC11790172; doi:10.1371/journal.pone.0318452)
Supplement: S2 Appendix — (DOCX) [file pone.0318452.s002.docx]

**S2 Appendix. Data set of patents diagnosed with Respiratory Epithelial Adenomatoid Hamartomas**

| Patient No. | **Laterality** | Age at operation | Gender | **Associated inflammatory polyp** | Symptom duration | Previous surgery | Asthma | Serum Eosinophil (%) | Serum Total IgE | JESREC score | Recurrence | Follow up period (days) |
| --- | --- | --- | --- | --- | --- | --- | --- | --- | --- | --- | --- | --- |
| 1 | Unilateral | 22 | M | No | >6mo | No | No | 6.4 | 93.7 | 8 | No | 199 |
| 2 | Unilateral | 41 | M | No | >6mo | Yes | No | 2.1 | 364 | 6 | No | 156 |
| 3 | Unilateral | 44 | M | No | NA | No | No | 0.6 | NA | NA | No | NA |
| 4 | Unilateral | 45 | M | No | <6mo | No | No | 3.1 | 10.5 | 4 | No | 481 |
| 5 | Unilateral | 47 | M | No | >6mo | No | No | 2.5 | 17.4 | 4 | No | 25 |
| 6 | Bilateral | 47 | M | No | >6mo | Yes | No | 6.4 | NA | 15 | No | 25 |
| 7 | Unilateral | 51 | M | No | NA | No | No | 0.3 | 283 | NA | No | NA |
| 8 | Unilateral | 61 | M | No | <6mo | No | No | 2.9 | NA | 4 | No | 363 |
| 9 | Unilateral | 64 | M | No | <6mo | No | No | 0.7 | NA | 0 | No | 193 |
| 10 | Unilateral | 45 | F | No | >6mo | Yes | No | 4 | NA | 4 | No | 38 |
| 11 | Bilateral | 34 | M | Yes | >6mo | Yes | Yes | 7.9 | NA | 15 | No | 313 |
| 12 | Bilateral | 40 | M | Yes | >6mo | No | No | 3.8 | 17.7 | 11 | No | 211 |
| 13 | Unilateral | 41 | M | Yes | >6mo | Yes | No | 5.2 | 78.7 | 10 | No | 172 |
| 14 | Unilateral | 44 | M | Yes | >6mo | No | No | 0.3 | 2.97 | 4 | No | 24 |
| 15 | Bilateral | 50 | M | Yes | >6mo | Yes | No | 0.6 | 38.2 | 7 | No | 107 |
| 16 | Bilateral | 67 | M | Yes | >6mo | Yes | Yes | 18 | NA | 17 | No | 29 |
| 17 | Bilateral | 37 | F | Yes | >6mo | Yes | Yes | 22 | NA | 17 | No | 63 |
| 18 | Bilateral | 61 | F | Yes | >6mo | Yes | Yes | 9.1 | NA | 15 | No | 4138 |
| 19 | Bilateral | 66 | F | Yes | >6mo | Yes | Yes | 1.1 | 87.2 | 7 | No | 34 |
| 20 | Unilateral | 42 | M | Yes | >6mo | Yes | No | 9.3 | 425 | 15 | No | 107 |
| 21 | Bilateral | 57 | M | Yes | >6mo | Yes | Yes | 11.2 | 156 | 17 | No | 110 |

IgE, Immunoglobulin E; JESREC score, Japanese Epidemiological Survey of Refractory Eosinophilic Chronic Rhinosinusitis score; mo, months; NA, Not Available
