# Supplementary material for: Link between bilaterality and type 2 inflammation in respiratory epithelial adenomatoid hamartomas: A two-center retrospective cohort study
Source: PLoS One. 2025 Feb 3;20(2):e0318452. doi: 10.1371/journal.pone.0318452 (PMC11790172; doi:10.1371/journal.pone.0318452)
Supplement: Attachment — Submitted filename: Response to Reviewers.docx [file pone.0318452.s003.docx]

------------------------------------------------------------------------------------------------------

**Point-by-point response**

------------------------------------------------------------------------------------------------------

**EDITOR**

This manuscript must be consistently edited either for English and mistakes contained in it.

**ANSWER:**

We appreciate your generous comments on improving this manuscript. We revised the manuscript to ensure proper English editing and corrected the identified mistakes. The parts edited with proper English are highlighted in blue.

------------------------------------------------------------------------------------------------------

**EDITOR**

In addition, the number of cases is considerably low and this must be written in the Limitations at the end of the Discussion in a **separate subheading**.

**ANSWER:**

We have updated a "Limitations" subheading at the end of the Discussion section explicitly stating the impact of the small sample size. Thank you for your insightful comment.

**CORRECTION:**

**Discussion (Page 11, Line 211-221)**

**Limitations**

This study has several limitations, primarily due to its retrospective design and small sample size of 21 patients. The small number of cases precluded formal sample size calculations and may limit the statistical power of our findings. However, it is essential to note that REAH remains an underdiagnosed and rare entity in clinical practice, making large cohort studies challenging. Despite these limitations, our series of 21 cases represents a meaningful contribution to the current literature on REAH, particularly regarding the characterization of bilateral versus unilateral disease patterns. Future prospective studies, including a larger cohort based on the origin of REAH, would help to further validate our findings, investigate the pathogenesis of REAH, and explore effective diagnostic methods and treatments based on its entity.

------------------------------------------------------------------------------------------------------

**EDITOR**
This study is based on a small sample size that greatly limits the study.
This must be clearly written in the Limitations of the study as a subheading before the Conclusions.

**ANSWER:**

Thank you for raising this important point. We have updated a "Limitations" subheading at the end of the Discussion section explicitly stating the impact of the small sample size.

**CORRECTION:**

**Discussion (Page 11, Line 211-221)**

**Limitations**

This study has several limitations, primarily due to its retrospective design and small sample size of 21 patients. The small number of cases precluded formal sample size calculations and may limit the statistical power of our findings. However, it is essential to note that REAH remains an underdiagnosed and rare entity in clinical practice, making large cohort studies challenging. Despite these limitations, our series of 21 cases represents a meaningful contribution to the current literature on REAH, particularly regarding the characterization of bilateral versus unilateral disease patterns. Future prospective studies, including a larger cohort based on the origin of REAH, would help to further validate our findings, investigate the pathogenesis of REAH, and explore effective diagnostic methods and treatments based on its entity.

------------------------------------------------------------------------------------------------------

**EDITOR**

In addition, the manuscript is plenty of mistakes either of language and content, as highlighted by reviewer #2.

**ANSWER:**

We appreciate your generous comments to improve this manuscript. We thoroughly revised the manuscript to correct all language and content issues highlighted by Reviewer #2.

------------------------------------------------------------------------------------------------------

**EDITOR**

The Table if not substituted by a Figure must change position.

**ANSWER:**

Thank you for your thoughtful comment. We repositioned Table 1 appropriately within the text for clarity and ensured it aligned with the surrounding discussion.

**CORRECTION:**

**Results (Page 8, line 142-146)**

**Table 1. Demographics of enrolled subjects**

|  | **REAH_bi (n=11)** | **REAH_uni (n=10)** | **p-value** |
| --- | --- | --- | --- |
| Age (years, mean ± SD) | 49.27 ± 11.78 | 43.40 ± 15.41 | 0.336 |
| Gender (Male, n, %) | 8 (72.7) | 9 (90.0) | 0.586 |
| Associated inflammatory polyp (n, %) | 10 (90.9) | 2 (20.0) | **0.002** |
| Symptom duration (>6months, n, %) | 11 (100.0) | 5 (62.5) | 0.058 |
| Previous surgery (n, %) | 10 (90.9) | 2 (20.0) | **0.002** |
| Asthma (n, %) | 6 (54.5) | 0 (0) | **0.012** |
| Serum Eosinophil (%, mean ± SD) | 8.32 ± 6.82 | 2.60 ± 2.15 | **0.021** |
| Serum Total immunoglobulin E | 177.59 ± 158.17 | 81.05 ± 105.90 | 0.231 |
| JESREC score (mean ± SD) | 12.91 ± 4.35 | 4.75 ± 3.01 | **<0.001** |
| Recurrence | - | - | - |
| Follow-up (months, mean±SD) | 16.04 ± 40.53 | 6.23 ± 5.53 | 0.509 |

SD, standard deviation; REAH; Respiratory epithelial adenomatoid hamartoma, REAH_bi; a group of patients with REAH originating from the bilateral superior septum or olfactory cleft, REAH_uni; a group of patients with REAH originating from unilateral sinonasal anatomy apart from the superior septum or olfactory cleft

------------------------------------------------------------------------------------------------------

**EDITOR**

The Discussion must not contain Results.

**ANSWER:**

We appreciate your valuable feedback. We moved all result-related content from the Discussion to the Results section to adhere to the correct manuscript structure.

**CORRECTION:**

**Results (Page 8, line 153-166)**

Endoscopic findings demonstrated a significantly higher association of inflammatory polyposis in REAH_bi compared to REAH_uni (Fig 2). Pathological review indicated no diagnostic differences between the groups (Fig 3).

**Figure 2.** Nasal endoscopic images.

REAH_bi (A) demonstrates indistinct boundaries between the lesion and inflammatory polyposis, whereas REAH_uni (B) shows a smooth mucosa-covered polypoid mass on the posterior nasal septum without other nasal lesions.

**Figure 3.** Microscopic findings of REAH

Both REAH_bi (A, B) and REAH_uni (C, D) display similar histological features, including glandular proliferation of pseudostratified ciliated respiratory epithelium and edematous stroma with scattered inflammatory cells (Hematoxylin and Eosin, magnification A: x100, B: x200, C: x100, D: x200).

------------------------------------------------------------------------------------------------------

**EDITOR**

The English requires a deep revision

**ANSWER:**

We appreciate your comment. We conducted a thorough English revision of the manuscript using a professional editing service. The revised parts are highlighted in blue.

------------------------------------------------------------------------------------------------------

**REVIEWER #1**

In the study, assuming that REAH can be located in different places in the sinonasal anatomy and that its clinical features may vary depending on the region of origin, a comprehensive study examining the pathologically confirmed clinical REAH series was conducted to investigate their potential relationship with clinical features indicative of type 2 chronic rhinosinusitis.

I suggest the authors to explain the calculation for sample size (G-power analysis) in the Methods section.

**ANSWER:**

Thank you for your insightful feedback and for the opportunity to further clarify our study methodology. Regarding your request for a G-Power analysis for sample size calculation, we would like to explain why such an analysis was not feasible in this context.

Respiratory epithelial adenomatoid hamartoma (REAH) is an underdiagnosed pathological entity, with limited cases reported in the literature and no comprehensive systematic reviews available to provide reliable estimates for effect sizes or prevalence.(reference) Our study is based on a retrospective review of all available cases treated and pathologically confirmed at our center, one of the largest tertiary referral hospitals in South Korea, over a 15-year period (November 2008 to November 2023), comprising 21 patients. Despite our institution's status as a major tertiary referral center, this limited number of cases reflects both the rarity of REAH and potential underdiagnosis due to limited awareness among pathologists. This constitutes the entire sample of this rare disease entity from our institution. Despite our efforts to include as many cases as possible, we kindly ask reviewers to understand the limited sample size in this study, which reflects the inherent rarity of this condition.

Given the rarity of REAH and the absence of prior systematic data, there are several challenges in applying G-Power analysis for this study:

1. No Established Effect Size

- G-Power calculations require an estimated effect size, typically derived from prior studies or systematic reviews. (ref.) In the absence of such data for REAH, any assumed effect size would be speculative and potentially misleading.

2. Retrospective Nature

- Our study reflects a complete retrospective cohort of patients treated within a specific timeframe, making it impractical to predefine or adjust the sample size based on power calculations.

3. Rarity of Disease

- REAH is an underdiagnosed disease entity, which inherently limits sample sizes, even at specialized centers. As such, the 21 cases represent not a subset but the full population available for study, making external sample size calculations inapplicable.

To address this, we have explicitly emphasized the scarcity of our sample size and the unavailability of performing G-Power analysis in the Limitations section of the Discussion. This addition acknowledges the inherent constraints of studying a rare disease and reinforces the need for future multicenter studies or systematic reviews to validate and expand upon our findings. We sincerely appreciate your valuable feedback to enhance the quality of our manuscript.

(Reference)

Davison WL, Pearlman AN, Donatelli LA, Conley LM. Respiratory epithelial adenomatoid hamartomas: An increasingly common diagnosis in the setting of nasal polyps. Am J Rhinol Allergy. 2016; 30: 139-146. https://doi.org/10.2500/ajra.2016.30.4338 PMID: 27456590

Issa MJA, Oliveira VRR, Nunes FB, Vasconcelos LOG, Souza LFB, Cherobin GB, et al. Prevalence of respiratory epithelial adenomatoid hamartomas (REAH) associated with nasal polyposis: an epidemiological study - how to diagnose. Braz J Otorhinolaryngol. 2022; 88 Suppl 5: S57-S62. https://doi.org/10.1016/j.bjorl.2021.09.009 PMID: 34844870

Lima NB, Jankowski R, Georgel T, Grignon B, Guillemin F, Vignaud JM. Respiratory adenomatoid hamartoma must be suspected on CT-scan enlargement of the olfactory clefts. Rhinology. 2006; 44: 264-269. PMID: 17216743

**CORRECTION:**

**Discussion (Page 11, Line 211-221)**

**Limitations**

This study has several limitations, primarily due to its retrospective design and small sample size of 21 patients. The small number of cases precluded formal sample size calculations and may limit the statistical power of our findings. However, it is important to note that REAH remains an underdiagnosed and rare entity in clinical practice, making large cohort studies challenging. Despite these limitations, our series of 21 cases represents a meaningful contribution to the current literature on REAH, particularly regarding the characterization of bilateral versus unilateral disease patterns. Future prospective studies, including a larger cohort based on the origin of REAH, would help to further validate our findings, investigate the pathogenesis of REAH, and explore effective diagnostic methods and treatments based on its entity.

------------------------------------------------------------------------------------------------------

**REVIEWER #2**

Comments and Suggestions for Authors

This retrospective study discussed the findings of respiratory epithelial adenomatoid hamartoma (REAH) and found that bilateral REAH exhibited type 2 inflammatory features more commonly compared to unilateral REAH. Since REAH is a rare disease often masked by other clinical features, such as nasal polyps, findings from this study provide a new perspective that can aid in the diagnosis of REAH. Also mentioned in the manuscript, the small sample size is one of the limitations of the study. Overall, the manuscript is well-written and easy to understand.

There are a few comments as below:
1. Abstract: (line 41) “…we report our treatment experience…”. The routine treatment for REAH is surgery. In the manuscript, treatment is not the main focus; the results and discussion are about the patients’ clinical history and characteristics. Suggest to add details of the treatment given or to edit the sentence.

**ANSWER:**

We are grateful for your insightful comments and recommendation to refine our work. We revised the abstract to better reflect the study focus and avoid overemphasis on treatment. The updated sentence emphasizes the clinical experience and disease characteristics.

**CORRECTION:**

**Abstract (Page 3, line 38-40)**

This study analyzes the clinical features of surgically treated sinonasal REAH and investigates disease characteristics based on bilaterality, particularly in relation to type 2 chronic rhinosinusitis.

------------------------------------------------------------------------------------------------------

**REVIEWER #2**

2. Materials and methods: for the date format, suggest spelling the month. For example, 1 November 2008 or November 1, 2008.

**ANSWER:**

We appreciate your valuable feedback. We adjusted the date format throughout the manuscript to spell out the month consistently.

**CORRECTION:**

**Materials and Methods (Page 6, line 102-105)**

We conducted a retrospective chart review of records between November 1, 2008, and November 1, 2023, involving patients diagnosed with REAH.

**Materials and Methods (Page 6, line 113-115)**

Data was collected and curated from July 15, 2022, to May 14, 2023, and updated between November 1, 2023, and April 1, 2024.

------------------------------------------------------------------------------------------------------

**REVIEWER #2**

3. Results: (page 8) Table 1 seems to be located in the middle of the text, not at the end of the paragraph. Suggest to check the format and text alignment to avoid confusion.

**ANSWER:**

Thank you for your careful review. We repositioned Table 1 to avoid it being placed mid-paragraph and ensured proper text alignment.

**CORRECTION:**

**Results (Page 8, line 142-146)**

**Table 1. Demographics of enrolled subjects**

|  | **REAH_bi (n=11)** | **REAH_uni (n=10)** | **p-value** |
| --- | --- | --- | --- |
| Age (years, mean ± SD) | 49.27 ± 11.78 | 43.40 ± 15.41 | 0.336 |
| Gender (Male, n, %) | 8 (72.7) | 9 (90.0) | 0.586 |
| Associated inflammatory polyp (n, %) | 10 (90.9) | 2 (20.0) | **0.002** |
| Symptom duration (>6months, n, %) | 11 (100.0) | 5 (62.5) | 0.058 |
| Previous surgery (n, %) | 10 (90.9) | 2 (20.0) | **0.002** |
| Asthma (n, %) | 6 (54.5) | 0 (0) | **0.012** |
| Serum Eosinophil (%, mean ± SD) | 8.32 ± 6.82 | 2.60 ± 2.15 | **0.021** |
| Serum Total immunoglobulin E | 177.59 ± 158.17 | 81.05 ± 105.90 | 0.231 |
| JESREC score (mean ± SD) | 12.91 ± 4.35 | 4.75 ± 3.01 | **<0.001** |
| Recurrence | - | - | - |
| Follow-up (months, mean±SD) | 16.04 ± 40.53 | 6.23 ± 5.53 | 0.509 |

SD, standard deviation; REAH; Respiratory epithelial adenomatoid hamartoma, REAH_bi; a group of patients with REAH originating from the bilateral superior septum or olfactory cleft, REAH_uni; a group of patients with REAH originating from unilateral sinonasal anatomy apart from the superior septum or olfactory cleft

------------------------------------------------------------------------------------------------------

**REVIEWER #2**

4. Discussion: (line 193 – 209). These are the results of endoscopic examination and microscopic appearance, which are not covered in the results section. Suggest to move it to the results.

**ANSWER:**

Thank you for your thoughtful comments. We relocated the endoscopic and microscopic findings content from the Discussion to the Results section.

**CORRECTION:**

**Results (Page 8, line 153-166)**

Endoscopic findings demonstrated a significantly higher association of inflammatory polyposis in REAH_bi compared to REAH_uni (Fig 2). Pathological review indicated no diagnostic differences between the groups (Fig 3).

**Figure 2.** Nasal endoscopic images.

REAH_bi (A) demonstrates indistinct boundaries between the lesion and inflammatory polyposis, whereas REAH_uni (B) shows a smooth mucosa-covered polypoid mass on the posterior nasal septum without other nasal lesions.

**Figure 3.** Microscopic findings of REAH

Both REAH_bi (A, B) and REAH_uni (C, D) display similar histological features, including glandular proliferation of pseudostratified ciliated respiratory epithelium and edematous stroma with scattered inflammatory cells (Hematoxylin and Eosin, magnification A: x100, B: x200, C: x100, D: x200).

------------------------------------------------------------------------------------------------------

**REVIEWER #2**
5. References:
i. (line 83, introduction) Reference no.13 described the details of immunohistochemistry findings of sinonasal adenocarcinomas, which are associated with REAH. However, the study did not include a genotypic or allelic study, hence not suitable to be referred for allelic loss.

**ANSWER:**

We appreciate your time and effort in providing detailed feedback for the improvement of this manuscript. Following your comment, we have revised the text to more accurately reflect the findings from reference 13, specifically highlighting the immunohistochemical evidence of the association between REAH and low-grade tubular sinonasal adenocarcinomas, while maintaining the separate discussion of allelic loss with reference 12.

**CORRECTION:**

**Introduction (Page 5, line 80-83)**

Additionally, unusually high allelic loss has been observed in polypoid sinus tissue [12]. Immunohistochemical analyses of low-grade tubular sinonasal adenocarcinomas associated with REAH have revealed shared characteristics, further supporting a potential neoplastic relationship [13].

------------------------------------------------------------------------------------------------------

**REVIEWER #2**

ii. (line 173, discussion) Reference no.3: In the article by Issa et al., 47.4% of the patients with polyposis were later confirmed to have REAH. Suggest to revise the number or sentence.

**ANSWER:**

Thank you for pointing out this numerical discrepancy. We have corrected the percentage from 50% to 47.4% to accurately reflect the findings reported in reference 3 by Issa et al. This ensures a precise representation of the cited data.

**CORRECTION:**

**Discussion (Page 9, line 179-181)**

Issa *et al.* demonstrated that 47.4% of 114 patients with olfactory cleft enlargement, initially treated for chronic rhinosinusitis with nasal polyps, were later histologically confirmed to have REAH [3].

------------------------------------------------------------------------------------------------------

We thank reviewers and editors for their invaluable comments.

Best regards,

Yong Gi Jung
